# Abnormal Levels of Liver Enzymes and Hepatotoxicity in HIV-Positive, TB, and HIV/TB-Coinfected Patients on Treatment in Fako Division, Southwest Region of Cameroon

**DOI:** 10.1155/2020/9631731

**Published:** 2020-05-12

**Authors:** Jude Eteneneng Enoh, Frederick Nchang Cho, Faustin Pascal Manfo, Simon Eyongabane Ako, Eric Achidi Akum

**Affiliations:** ^1^Department of Medical Laboratory Science, Faculty of Health Sciences, University of Buea, P.O. Box 63, Buea, Cameroon; ^2^Infectious Disease Laboratory, Faculty of Health Sciences, University of Buea, P.O. Box 63, Buea, Cameroon; ^3^Laboratory of Endocrinology and Radio-Elements, Medical Research Centre, Institute of Medical Research and Medicinal Plants Studies, P.O. Box 13033 Yaoundé, Cameroon; ^4^Central Africa Network on Tuberculosis, HIV/AIDS and Malaria-European and Developing Countries Clinical Trials Partnership (CANTAM-EDCTP), Congo; ^5^Department of Biochemistry and Molecular Biology, Faculty of Science, University of Buea, P.O. Box 63, Buea, Cameroon; ^6^Medical Research and Bacteriology Laboratory, Faculty of Health Sciences, University of Buea, P.O. Box 63, Buea, Cameroon

## Abstract

Hepatotoxicity is historically the 3^rd^ most common reason for drug withdrawal and toxicity-related discontinuation of treatment. This study was aimed at determining the incidence and the onset of hepatotoxicity and at evaluating the relationship of some risk factors for hepatotoxicity among Human Immunodeficiency Virus- (HIV-) positive, tuberculosis (TB), and HIV/TB patients on treatment. This was a prospective follow-up study involving 125 participants from the HIV/AIDS and TB treatment centres in three hospitals in Fako Division of Cameroon. These TB and HIV patients were initiated on RHEZ (R = Rifampicin, H = Isoniazid, E = Ethambutol, and P = Pyrazinamide) and TELE (efavirenz/tenofovir/lamivudine), respectively, and followed up for 12 weeks between September 2018 and November 2019. The levels of liver enzymes (transaminases, gamma-glutamyltransferase, alkaline phosphatase, and unconjugated/total bilirubin) were measured spectrophotometrically using serum. The Chi-squared (*χ*^2^) test was used to assess the association between risk factors and hepatotoxicity, while the Kaplan-Meier survival analysis with the log-rank test was used to determine the occurrence of hepatotoxicity in the different groups. We followed the general study population for a total person time of 6580 person-days, with an incidence rate and cumulative incidence of 8 cases per 1000 person-days (53/6580 person-days) and 42.4% (53/125), respectively (95% confidence interval), recorded after 12 weeks of follow-up of all the participants. The onset of hepatotoxicity in the total study population was statistically significant (*χ*^2^ = 9.5334; *p* = 0.022979; CI = 95%), with the majority observed at week eight of follow-up. Also, the incidence rate and cumulative incidence of hepatotoxicity with respect to HIV/AIDS, TB, and HIV/TB patients, respectively, at 95% confidence interval were: 8 cases per 1000 person-days (32/3843 person-days) and 32/76 (42.1%), 6 cases per 1000 person-days (12/1932 person-days) and 12/32 (37.5%), and 11 cases per 1000 person-days (9/805 person-days) and 9/17 (52.9%). This study shows that the incidence rate and cumulative incidence of hepatotoxicity in HIV/AIDS, TB, and HIV/TB patients on treatment were high in Fako Division, Cameroon. Also, it is very important to check these patients' liver function especially within the first 12 weeks of treatment.

## 1. Introduction

Acquired immune deficiency syndrome (AIDS) is a chronic immunosuppressed and potentially life-threatening state caused by a retrovirus (called Human Immunodeficiency Virus (HIV)) [1, 2]. In records, more than 70 million people have been infected with HIV, with about 35 million HIV-associated deaths [3]. Globally, 37.9 million (31.1–43.9 million) people living with HIV (PLWH) were recorded at the end of 2017. By 2018, in Cameroon, a total of 540,000 people were living with HIV; HIV prevalence among adults (15–49 years) was 3.6%; newly infected with HIV was 23,000, 18,000 people died from an AIDS-related illness, and 81% of all people living with HIV were on treatment [4]. In 2017, 50.7% of people living with HIV and tuberculosis were on treatment for both diseases, compared to 39.5% in 2015 [4].

In 2018, TB alone was responsible for about 1.2 million deaths (range, 1.1–1.3 million) and an additional 300,000 deaths (range, 266,000–335,000) among HIV-positive individuals [3]. Globally, an estimated 10.0 million people (range, 9.0–11.1 million) developed TB disease in 2017: 5.8 million men, 3.2 million women, and 1.0 million children. According to the World Health Organization (WHO) country report of Cameroon in 2017, the National TB Control Program reported 24,527 new and relapse cases of TB and 31% were positive for HIV [5].

DOTS (Directly Observed Treatment, Short-Course) is the name given to the tuberculosis control strategy recommended by the World Health Organization [5, 6]. Currently, more than twenty drugs are known to be used for the treatment of TB, and although these drugs efficiently combat the microorganism, they can result in undesirable side effects, due either to the active principle itself or to its metabolites. Side effects, principally the most severe, are related to higher rates of treatment abandonment [7–9].

The complexity of managing these diseases could be increased due to the existence of numerous drug-drug interactions between TB and HIV drugs [10].

Among other diseases, liver disease with elevated liver enzymes such as hepatotoxicity and drug-induced liver injury (DILI) has emerged as the common non-AIDS- and non-TB-related cause of death among HIV and TB patients, with about 14-18% deaths recorded in HIV patients alone [11]. These elevated liver enzymes range from mild to cirrhosis and end-stage liver diseases with its complications that account for almost half of the deaths among HIV patients [11]. Drug-induced hepatotoxicity (DIH), that is, an abnormal level of liver enzymes in patients on treatment, is becoming one of the major concerns in medical practice. Although abnormal levels of liver enzymes, hepatotoxicity, or drug-induced liver injury (DILI) is relatively uncommon, it is still the leading cause of nonadherence to treatment, acute liver failure, and a significant reason for liver transplantation [12, 13]. The general population and healthcare system have an estimated incidence of 14–19 per 100,000 inhabitants and 30–33 per 100,000 persons, respectively, of DILI [14, 15]. This reported incidence of DILI varies among various populations and drugs used, suggesting that drug properties also have a role in DILI risk determination [14, 15]. The host factors of patients could also be the main cause for this elevated liver enzymes as well as DILI, since drugs with DIH or DILI potential cause liver injury only in a small portion of patients [13]. Elevated levels of serum activity of transaminases are commonly measured and used among other enzymes (bilirubin, albumin, and alkaline phosphatase) to determine liver cell injury. Many factors such as medications, alcoholism, host factors, and geographical area are among the risk factors associated with elevated liver enzymes as well as hepatotoxicity.

The aims of this study were to (1) determine the incidence and prevalence of hepatotoxicity among patients and (2) to evaluate the relationship of possible risk factors associated with hepatotoxicity in HIV/AIDS, TB, and HIV/TB patients on treatment.

## 2. Material and Methods

### 2.1. Study Area

This study was conducted in HIV/AIDS and tuberculosis treatment centres of three selected hospitals: Limbe Regional Hospital (LRH), Buea Regional Hospital Annex (BRHA), and Mutengene Baptist Hospital (MBH), in Fako Division, Southwest Region of Cameroon, between September 2018 and November 2019. This study area covers one of the biggest HIV/TB treatment centres, which is among the 223 diagnostic and treatment centres for tuberculosis patients coordinated by the Cameroon National Tuberculosis Control Program.

### 2.2. Study Design and Population

It was a prospective cohort hospital-based study. The study population was naïve HIV/AIDS, naïve TB, and HIV/TB-coinfected patients, who accepted to be put on treatment. We enrolled 183 participants consecutively, from which the 125 involved in the study were selected based on the inclusion and exclusion criteria. The expected sample size was 173 using 13.6% prevalence of abnormal liver enzymes among HIV-infected patients on ATD taken from a similar setting [16], 95% confidence interval, and the margin of error 0.05.

We had three groups from the study participants: HIV/AIDS, TB, and TB/HIV.

*Inclusion criteria*: patients aged ≥15 years, newly diagnosed HIV/AIDS patients who accepted to be initiated on HAART, and patients newly diagnosed with TB with positive sputum smear.

*Exclusion criteria*: all those who had abnormal levels of the renal function test and liver function test (greater than two times the ULN) and hepatitis before the start of treatment, pregnant and lactating women, patients receiving any other hepatotoxic drugs parallel with HAART and antituberculosis drugs (ATD), and patients who are not willing to give useful and truthful information.

The participants were on the first-line regimen of HAART (TDF, 3TC, and EFZ) or ATD (INH, PZA, RIF, and EMB), and also, all the participants on HAART were on cotrimoxazole and TB prophylaxis (INH).

### 2.3. Data Collection

Naïve HIV/AIDS and TB patients reporting for scheduled and unscheduled treatment and check-up visits were enrolled consecutively.

We reviewed hospital records of consenting participants for medical history focus on the inclusion and exclusion criteria. A standardised questionnaire was administered to all the participants enrolled. The questions gave information on sociodemographic characteristics and clinical and epidemiological data including gender, age, alcohol abuse, smoking, TB regimen, HIV regimen, ethnicity, antioxidant food, other diseases, and concomitant use of other medications.

### 2.4. Patient Monitoring

Also, some hepatotoxicity clinical signs such as fever, nausea, vomiting, and tiredness were monitored as well as episodes of malaria and opportunistic infections. The participants were followed for 12 weeks, and a morbidity questionnaire was also administered before sample collection at one week, four weeks, eight weeks, and twelve weeks after treatment initiation. Liver enzymes were analysed every time samples were collected for ALT (Alanine Transaminase), AST (Aspartate Transaminase), GGT (gamma-glutamyltransferase), ALP (alkaline phosphatase), and total and direct bilirubin.

Participants were assigned unique identification numbers that were on the questionnaires, tubes of sample collection, and result form.

### 2.5. Sample Collection

About 2 ml of venous blood was collected by venipuncture under a sterile condition into 5 ml vacutainer dry test tubes. The samples were allowed to clot by leaving it undisturbed at room temperature and then centrifuged later for 5 minutes at 3000 rpm, and the supernatant (serum) was collected into an Eppendorf tube and stored at -8°C until batch laboratory analysis.

### 2.6. Laboratory Analysis

The laboratory tests are liver function tests (LFT), renal function tests (RFT), lipid profile, and hepatitis B and C. The laboratory analyses were done according to the same clinical schedule in all three groups of patients at the Infectious Disease Laboratory-FHS and the Saint Albert the Great Clinic, Routine Laboratory.

Hepatotoxicity was defined following the International Consensus Criteria and also on previous studies [17, 18]:

- There is an increase in serum liver enzymes (AST, ALT, ALP, and GGT) greater than three times the upper limit of normal (ULN) levels and bilirubin twice the ULN after treatment
- In cases with an abnormal level of basal AST or ALT before treatment initiation, the level(s) after the start of treatment was double the basal level

The severity of the liver injury was indicated by category (graded) based on various enzyme levels (Grade 1, Grade 2, Grade 3, and Grade 4) [19–21].

Also, the ratio ALT : ALP was used to decide the type of liver damage by hepatotoxins [21]:

- The ratio ALT : ALP ≥ 5 = hepatocellular damage
- The ratio ALT : ALP ≤ 2 = cholestatic liver damage
- The ratio ALT : ALP between two and five = mixed types of liver damage

### 2.7. Statistical Analysis

Data were entered into and analysed with IBM-SPSS Statistics 21.0 for Windows (IBM-SPSS Corp., Chicago, USA). The Chi-squared (*χ*^2^) test was used to compare sociodemographic characteristics and some possible risk factors to identify significant correlation with hepatotoxicity. Kaplan-Meier survival analysis with the log-rank test was used to compare the occurrence and the time of occurrence of hepatotoxicity in the different study groups; *p* values less than 0.05 were considered significant with a confidence interval of 95%.

### 2.8. Ethical Considerations

The ethical approval of the study was obtained from the Ethical Review Committee of the Faculty of Health Sciences Institutional Review Board of the University of Buea (Ref: 2018/153/UB/SG/IRG/FHS) and Cameroon Baptist Convention Institutional Review Board (Ref: IRB2018-48). Administrative clearances from the Southwest Regional Delegation for Public Health and directors of the various hospitals concerned were obtained. Written informed consents were gotten from each patient.

## 3. Results

A total of 183 treatment-naïve TB, HIV, and/or TB/HIV patients were enrolled for the study. Out of the 183 patients, 125 who fit the inclusion and exclusion criteria were followed up for twelve weeks. Baseline sociodemographic characteristics for the patients stratified by the treatment group are presented in Table 1.

### 3.1. Incidence of Hepatotoxicity

Of the 125 patients with no sign of abnormal levels of liver enzymes at initiation, and who fits both inclusion and exclusion criteria, we successfully followed them for a total person time of 6580 person-days. The incidence rate and incidence proportion were 53/6580 person-days (8 cases per 1000 person-days) and 53/125 (42.4%), respectively, at the end of the study period. Also, the following frequency (%), 20/125 (16.0%), 8/125 (6.40%), 21/125 (16.8%), and 4/125 (3.2%), of hepatotoxicity was observed based on the time of onset that is one, four, eight, and twelve weeks of follow-up, respectively, which was statistically significant (*χ*^2^ = 9.5334; *p* = 0.022979; CI = 95%).

**Figure 1:**
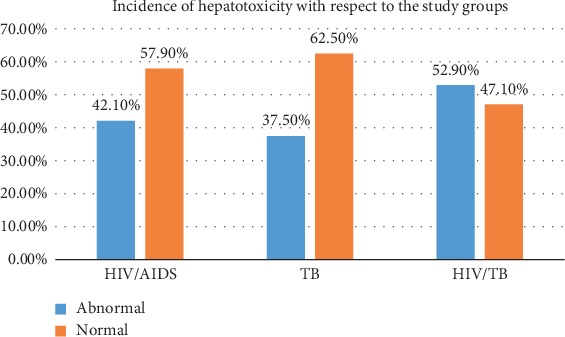
Cumulative Incidence of hepatotoxicity with respect to the study groups.

The observed cumulative incidence of hepatotoxicity with respect to the different study groups was 32/76 (42.1%), 12/32 (37.5%), and 9/17 (52.9%) in HIV/AIDS, TB, and HIV/TB patients, respectively (Figure 1).

Also, we observed the following incidence rates: 8 cases per 1000 person-days (32/3843 person-days), 6 cases per 1000 person-days (12/1932 person-days), and 11 cases per 1000 person-days (9/805 person-days) for HIV/AIDS, TB, and HIV/TB patients, respectively.

**Figure 2:**
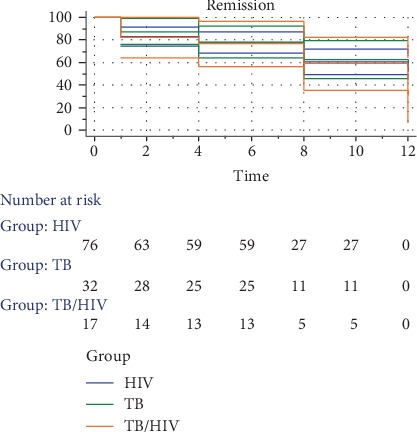
Kaplan-Meier survival curves with respect to the study groups.

Comparing the survival curves (log-rank test) of hepatotoxicity, we observed that there is no statistically significant difference in occurrence rates of the participants in the various groups and that the grouping has no significant influence on time of onset (*χ*^2^ = 0.8767; *p* = 0.6451; CI = 95%) (Figure 2).

The overall Survival Proportion (SP) and Standard Error (SE) with respect to the weeks of follow-up were SP = 0.840, SE = 0.0328; SP = 0.776, SE = 0.0373; SP = 0.608, SE = 0.0437; and SP = 0.551, SE = 0.0479 for weeks 1, 4, 8, and 12, respectively.

Looking at the association of some possible risk factors with hepatotoxicity, we recorded no statistically significant association between the risk factors and hepatotoxicity (Table 2).

### 3.2. Grading/Categorising Hepatotoxicity with respect to Weeks of Follow-Up

In regard to grading those with abnormal levels of liver enzymes (ALE), we observed the highest cumulative incidence 26.0% (20/77) of Grade 1 ALT elevation at week 12, 35.2% (44/125) of Grade 1 AST elevation at week 1, 20.0% (25/125) of Grade 1 GGT elevation at week 1, 15.2% (19/125) of Grade 1 ALP elevation at week 4, 35.00% of type 3 DILI (TBili) at week 12, and 16.8% (21/125) of liver injury (cholestatic) at week 8 (Table 3).

**Figure 3:**
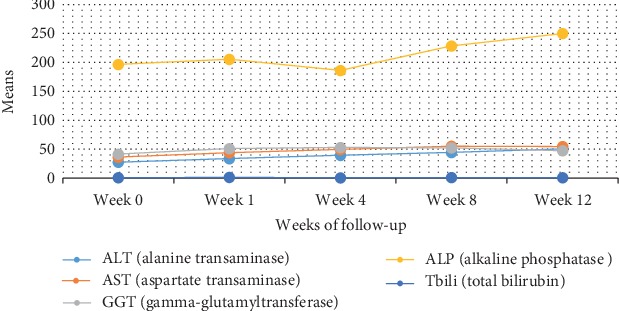
Means of liver enzymes with respect to weeks of follow-up.

Also, we recorded a steady increase in the means of the ALT and AST enzyme with respect to the weeks of follow-up. For GGT, the highest mean was observed at week four, and it starts decreasing. The mean of ALP had a drop at week four and after which we notice a steady increase at weeks 8 and 12 (Figure 3).

## 4. Discussion

Elevated levels of liver enzymes have been reported in several studies involving naïve HIV-positive and TB patients as well as those on treatment [13, 22, 23]. The results of our study then add more weight to these earlier studies about the fact that HAART and ATD have the potential to induce liver injury. This study follows up patient on HAART and ATD to illustrate possibilities of liver abnormalities due to the drug and other possible risk factors such as social life, age, and gender.

Majority of the participants had secondary and primary education or no level of education. This result shows that more is still needed when it comes to the sensitisation process about HIV/AIDS, TB, and infectious disease in general. This adds more light to the fact that literacy and especial health literacy can impact the prevention of diseases [24].

Our incidence rate and proportion of 8 cases per 1000 person-days and 42.4%, respectively, were quite higher, as compared to similar studies such as an incidence rate 14–19 per 100,000 in Europe reported by Einar [15] and incidence proportion of 30% in Brazil by Araújo-Mariz et al. [25], 8% in Ethiopia reported by Wondwossen et al. [18], 18.2% in Nigeria by Samson et al. [26], and 7.3% by McNeil et al. [27]. The variation in the incidence from study to study may be attributed to the differences in the individuality of the participants in the study population, indiscriminate use of drugs, and the definition criteria of hepatotoxicity [17–22].

Also, the high cumulative incidence of hepatotoxicity 52.9%, 42.1%, and 37.5% observed in HIV/TB, HIV/AIDS, and TB participants, respectively, could be a result of their combination therapy (HAART, RHEZ) in addition to cotrimoxazole and TB prophylaxis (INH) in HIV patients, since it has been reported by other studies the possible effect of cotrimoxazole [23] and Isoniazid [28] to liver injury. Likewise, the onset of hepatotoxicity with respect to the different groups was not statistically significant, indicating that the grouping had no effect on the time of occurrence, since the patients were on one or two common drugs (cotrimoxazole and INH) during the follow-up.

The onset of hepatotoxicity was statistically significantly associated (*χ*^2^ = 9.5334; *p* = 0.022979; *CI* = 0.05) with majority 16.0% and 16.8% hepatotoxicity recorded in weeks 1 and 8, respectively. In addition, this finding is consistent with another study by Wondwossen et al. [18], whose time interval for the start of hepatotoxicity after initiation of treatment was 13–58 days (median, 26 days). This result then buttresses the importance to monitor the liver's function during the early weeks in patients on treatment and could be helpful for very early detection of hepatotoxicity and improvement of management [17].

Furthermore, alcohol consumption has been shown to be an obvious risk factor for intrinsic hepatotoxicity [29], but in our study, alcohol consumption was not statistically significantly associated to hepatotoxicity which is line with similar findings by Wondwossen et al. [18].

Findings of this study showed no statistically significant association between gender and hepatotoxicity, which is similar to another study by Golemba et al. [30] but contrary to another previous study by Wondwossen et al. [18]. However, the cumulative incidence of hepatotoxicity observed in females (54.7%) was slightly higher than that in males (45.3%) which could be because of CYP3A4, a primary drug-metabolising enzyme which is expressed at a higher rate in women [12, 31].

In addition, our results showed that hepatotoxicity was more prevalent among participants within the age range ≥ 40 years which is in agreement with a similar study by Jennifer et al. [32]. Our findings also show that there was not an association of age and DILI, which is an agreement with the previous research by Wondwossen et al. [18].

Looking at disease burden as a risk factor, we recorded a frequency of 28.3% abnormal levels of liver enzymes in participants who were diagnosed of some opportunistic infections, and this could be a result of their current disease state. Diseases can change the pharmacokinetics of drugs and lead to unexpected effects such as an abnormal liver function or hepatotoxicity [33], and in this case, we were dealing with HIV/AIDS and TB patients who are immunocompromised; it looks more likely for such effect.

## 5. Strengths of the Study

The data used was collected by experienced scientists and health personnel, and the data was collected with a questionnaire and case report forms as well as by observations.

## 6. Limitations of the Study

A limitation to the study was that there was no close monitoring of the medications consumed during the three months of follow-up as this may have consequences on the liver.

## 7. Conclusion

This study shows that the incidence rate and cumulative incidence of hepatotoxicity inHIV/AIDS, TB, and HIV/TB patients on treatment are high in Fako Division, Southwest Region of Cameroon. Also, it is very important to check these patients' liver function especially within the first 12 weeks of treatment. With the nonassociation of some common risk factors such as alcohol with hepatotoxicity, these results then show other risk factors such as the drugs, or the patients' genetic constitution may likely be the cause. So, we recommend further studies precisely on the genetic aspect with hepatotoxicity, such as polymorphism of some key drug metabolism enzymes, since several reports have shown that personal factors are highly associated with hepatotoxicity compared to the drugs itself.

## Figures and Tables

**Table 1:** Baseline characteristics of the study population stratified by treatment groups.

Variable	Subclass	HIV (%)	TB (%)	TB/HIV (%)	Total (%)	*χ* ^2^	*p* value
Gender	Female	46 (60.5)	15 (46.9)	07 (41.2)	68 (54.4)	3.0787	0.214
	Male	30 (39.5)	17 (53.1)	10 (58.8)	57 (45.6)		

Age (years)	<40	34 (44.7)	22 (68.8)	6 (35.3)	62 (49.6)	6.8051	0.332
	≥40	42 (55.3)	10 (31.2)	11 (64.7)	63 (50.4)		
	Mean age (±SD)	39.54 ± 10.69	39.54 ± 10.69	39.54 ± 10.69	39.54 ± 10.69		

BMI	Underweight	04 (5.3)	05 (15.6)	01 (5.9)	10 (8.00)	7.4444	0.11418
	Normal	40 (52.6)	20 (62.5)	12 (70.6)	72 (57.6)		
	Overweight	32 (42.1)	07 (21.9)	04 (23.5)	43 (34.4)		
	Mean BMI (±SD)	24.32 ± 4.23	24.55 ± 4.35	24.32 ± 4.23	24.32 ± 4.23		

Education	NFE+primary	23 (30.3)	10 (31.2)	06 (35.3)	39 (31.2)	7.7285	0.102046
	Secondary	41 (53.9)	11 (34.4)	05 (29.4)	57 (45.6)		
	Tertiary	12 (15.8)	11 (34.4)	06 (35.3)	29 (23.2)		

Marital status	Married	28 (36.8)	11 (34.4)	06 (35.3)	45 (36.0)	0.0637	0.968
	Unmarried	48 (63.2)	21 (65.6)	11 (64.7)	80 (64.0)		

Occupation	Skilled	13 (17.1)	02 (6.3)	03 (17.6)	18 (12.8)	11.6556	0.070109
	Unskilled	54 (71.1)	22 (68.8)	12 (70.6)	88 (72.0)		
	Student	03 (3.9)	07 (21.8)	2 (11.8)	12 (9.60)		
	Unemployed	06 (7.9)	01 (3.1)	00 (0.00)	07 (5.60)		

WHO clinical stage	Stage 0	00 (0.0)	32 (100.0)	00 (0.00)	32 (25.6)		
	Stage 1	18 (23.7)	00 (0.00)	04 (23.5)	22 (17.6)		
	Stage 2	39 (51.3)	00 (0.00)	06 (35.3)	45 (36.0)		
	Stage 3	19 (25.0)	00 (0.00)	07 (41.2)	26 (20.8)		

BCG scar	Yes	44 (57.9)	20 (62.5)	09 (52.9)	73 (58.4)	0.4379	0.80335
	No	32 (42.1)	12 (37.5)	08 (47.1)	52 (41.6)		

LNE	Yes	0 (0.0)	0 (0.0)	2 (11.8)	2 (1.6)		
	No	76 (100.0)	32 (100.0)	15 (88.2)	123 (98.4)		

Type of TB	Pulmonary	00 (0.0)	27 (84.4)	16 (94.1)	43 (34.4)		
	Extrapulmonary	00 (0.0)	05 (15.6)	01 (5.9)	06 (4.8)		
	Negative	76 (100)	00 (0.0)	00 (00.0)	76 (60.8)		

OI	Yes	31 (40.8)	00 (0.0)	03 (17.6)	34 (27.2)		
	No	45 (59.2)	32 (100.0)	14 (82.4)	91 (72.8)		

Malaria	Yes	17 (22.4)	12 (37.5)	07 (41.2)	36 (28.8)	3.9842	0.136
	No	59 (77.6)	20 (62.5)	10 (58.8)	89 (71.2)		
	Total	*n* = 76	*n* = 32	*n* = 17	*N* = 125		

OI = opportunistic infection; NFE = no formal education.

**Table 2:** Association of hepatotoxicity with possible risk factors.

Variables		Abnormal liver enzymes		Total (%)	*χ* ^2^	*p* value
		Yes (%) *n* = 53	No (%) *n* = 72			
Gender	Male	24 (45.3)	44 (61.1)	68 (54.4)	3.0831	0.79108
	Female	29 (54.7)	28 (38.9)	57 (45.6)		

Age range	<40	26 (49.1)	36 (50.0)	62 (49.6)	0.0109	0.916969
	≥40	27 (50.9)	36 (50.0)	63 (50.4)		

Drug regimens	ATD	12 (22.6)	20 (27.8)	32 (25.6)	1.0908	0.57962
	HAART	32 (60.4)	44 (61.1)	76 (60.8)		
	ATD/HAART	9 (17.0)	8 (11.1)	17 (13.6)		

Malaria	Yes	16 (30.2)	21 (29.2)	37 (29.6)	0.0153	0.901552
	No	37 (69.8)	51 (70.8)	89 (71.2)		

Opportunistic infection	Yes	15 (28.3)	18 (25.0)	34 (26.4)	0.1352	0.7131
	No	38 (71.7)	54 (75.0)	92 (73.6)		

Alcoholism	Yes	13 (24.5)	17 (23.6)	30 (24.0)	0.8778	0.644749
	Ex-drinker	28 (52.8)	28 (38.9)	56 (44.8)		
	No	12 (22.7)	27 (37.5)	39 (31.2)		

Smoking	Yes	1 (1.9)	1 (1.4)	2 (1.60)	0.7927	0.672788
	Ex-smoker	6 (11.3)	5 (6.9)	11 (8.80)		
	No	46 (86.8)	66 (91.7)	112 (89.6)		

Total (%)		53 (42.4)	72 (57.6)	125 (100)		

**Table 3:** Different categories (grade) of hepatotoxicity with respect to weeks of follow-up.

Variable	Subclass	Baseline (%)	Week 1 (%)	Week 4 (%)	Week 8 (%)	Week 12 (%)
ALT levels	Normal	125 (100.0)	109 (87.2)	107 (85.6)	100 (80.0)	49 (63.6)
	Grade 1	—	13 (10.4)	14 (11.2)	15 (12.0)	20 (26.0)
	Grade 2	—	03 (2.4)	04 (3.2)	10 (8.0)	08 (10.4)
	Mean ALT (±SEM)	27.52 ± 1.73	33.72 ± 2.21	39.56 ± 2.22	44.41 ± 2.81	50.65 ± 3.83

AST levels	Normal	125 (100.0)	74 (59.2)	82 (65.6)	89 (71.2)	40 (52.0)
	Grade 1	—	44 (35.2)	34 (27.2)	18 (14.4)	27 (35.0)
	Grade 2	—	07 (5.6)	09 (7.2)	18 (14.4)	10 (8.0)
	Mean AST (±SEM)	36.40 ± 2.97	43.94 ± 2.66	49.64 ± 2.77	55.22 ± 3.28	54.64 ± 3.42

GGT levels	Normal	125 (100.0)	100 (80.0)	93 (74.4)	109 (87.2)	57 (74.0)
	Grade 1	—	18 (14.4)	25 (20.0)	09 (7.2)	19 (24.7)
	Grade 2	—	6 (4.8)	6 (4.8)	07 (5.6)	01 (1.3)
	Grade 3	—	1 (0.8)	1 (0.8)	—	—
	Mean GGT (±SEM)	41.56 ± 3.22	51.11 ± 3.66	53.04 ± 3.96	52.31 ± 3.25	47.45 ± 3.02

ALP levels	Normal	125 (100.0)	113 (90.4)	101 (80.8)	120 (96.0)	66 (85.7)
	Grade 1	—	11 (8.8)	19 (15.2)	05 (04.0)	11 (14.3)
	Grade 2	—	1 (0.8)	5 (4.0)	—	—
	Mean ALP (±SEM)	196.53 ± 11.29	205.47 ± 11.90	186.15 ± 12.23	228.53 ± 11.11	250.03 ± 15.00

TBili levels	Normal	125 (100.0)	99 (79.2)	111 (88.8)	97 (77.6)	59 (76.6)
	Grade 1	—	14 (11.2)	7 (5.6)	10 (8.0)	11 (14.3)
	Grade 2	—	3 (2.4)	5 (4.0)	13 (10.4)	07 (9.1)
	Grade 3	—	4 (3.2)	—	02 (1.6)	—
	Grade 4	—	5 (4.0)	2 (1.6)	03 (2.4)	—
	Mean TBili (±SEM)	0.78 ± 0.45	1.39 ± 0.31	0.64 ± 0.14	1.09 ± 0.20	0.71 ± 0.06

Type of liver injury	Normal	125 (100.0)	105 (84.0)	117 (93.6)	104 (83.2)	73 (94.8)
	Hepatocellular	—	—	—	—	—
	Cholestatic	—	20 (16.0)	08 (6.4)	21 (16.8)	04 (5.2)
	Mixed	—	—	—	—	—
	Mean *R* factor (±SEM)	0.25 ± 0.033	0.33 ± 0.089	0.42 ± 0.051	0.25 ± 0.019	0.23 ± 0.018

Alcohol-related LI	GGT: ALP < 2.5	125 (100.0)	116 (92.8)	113 (90.4)	121 (96.8)	77 (100.0)
	GGT: ALP > 2.5	0 (00.0)	09 (7.2)	12 (9.6)	04 (3.2)	0 (00.0)
	Total	125	125	125	125	77

## Data Availability

The data used was collected by experienced scientists and health personnel, and the data was collected with a questionnaire, case report forms as well as by observations.

## Abbreviations

95% CI: 95% confidence interval
HA: Health area
NFE: No formal education
*p* value: Significance value
SD: Standard deviation
*χ*^2^: Chi square
ALE: Abnormal liver enzymes
EFV: Efavirenz
EMB: Ethambutol
INH: Isoniazid
PCR: Polymerase chain reaction
PZA: Pyrazinamide
RHEZ: Rifampicin, Isoniazid, Ethambutol, and Pyrazinamide
RIF: Rifampicin
TELE: Tenofovir, Lamivudine, and Efavirenz
TB: Tuberculosis
TDF: Tenofovir
IRB-FHS: Institutional Review Board Faculty of Health Sciences
CBC-IRB: Cameroon Baptist Convention Institutional Review Board
MBH: Mutengene Baptist Hospital
LRH: Limbe Regional Hospital
BRHA: Buea Regional Hospital Annex.
